# Utilization of Wilms’ tumor 1 antigen in a panel for differential diagnosis of ovarian carcinomas

**DOI:** 10.4274/tjod.22220

**Published:** 2016-03-10

**Authors:** Dilek Şakirahmet Şen, Ayşe Filiz Gökmen Karasu, Melin Özgün Geçer, Nimet Karadayı, Elif Ablan Yamuç

**Affiliations:** 1 Lütfi Kırdar Research and Training Hospital, Department of Pathology, İstanbul, Turkey; 2 Bezmialem Vakıf University Faculty of Medicine, Department of Obstetrics and Gynecology, İstanbul, Turkey; 3 Bezmialem Vakıf University Faculty of Medicine, Department of Pathology, İstanbul, Turkey

**Keywords:** Wilms tumor 1, ovarian cancer, cytokeratin 7, cytokeratin 20

## Abstract

**Objective:**

Ovarian metasteses are often mistaken for primary adenocarcinoma. Studies conducted in recent years have focused on a search for an immunohistochemical marker to aid the differential diagnosis primary and metastatic ovarian carcinoma. Our study objective was to study the usefulness of Wilms tumor 1 (WT 1) antigen in this context.

**Materials and Methods:**

The study was conducted at the pathology clinic of Lütfi Kırdar Training and Research Hospital. Deparaffinated blocks of 40 epithelial ovarian tumors, 40 colon adenocarcinomas, and 35 cases of omentum metastases were studied. Cytokeratin 7 (CK 7), cytokeratin 20 (CK 20), and WT 1 were applied to all specimens.

**Results:**

All ovarian adenocarcinomas were stained with CK 7 (100%). Colorectal adenocarcinomas were stained positive with CK 20 in 87.5% of cases. Primary ovarian adenocarcinomas stained positive with WT 1 in 82.5% of the cases and none of the colorectal adenocarcinomas showed staining with WT 1 (0%).

**Conclusion:**

WT 1 can be used in conjuction with CK 7 in the differential diagnosis of ovarian carcinomas.

## INTRODUCTION

Ovarian tumors constitute approximately 6% of all malignancies and 30% of all gynecologic malignancies in women^(1)^. Ninety percent of ovarian tumors arise from the surface epithelium of the ovaries. Metastatic ovarian carcinomas make up 3-6% of ovarian malignancies and may originate primarily from genital or nongenital organs^(2)^. Endometrium, fallopian tubes, breasts, as well as gastrointestinal and hematopoietic tissue may be the location of primary tumors. Studies to date have examined patients presenting with ovarian masses; therefore, microscopic metastases have mostly been overlooked. In order to document the incidence of metastatic ovarian tumors, some researchers have comitted to performing autopsies. Fox and Langley^(3)^ performed autopsies on 272 women who died of malignancy and discovered that 4.4% of the cases involved ovarian metasteses. One difficulty in diagnosing metastatic ovarian carcinoma is that even after meticulous histologic examination, metasteses are often mistaken for primary adenocarcinoma or vice versa^(4,5,6,7,8,9)^.

Immunohistochemistry with cytokeratin (CK) labelling may be helpful in the differential diagnosis. CKs are intracellular proteins found widely in epithelial tissue. They are classified according to their pH and molecular weight. Expression of these CKs is frequently organ or tissue specific. CKs tend to remain stable when an epithelium undergoes malignant transformation. CK 7 is expressed in ovarian, lung and breast epithelia, but generally not in colon and prostate epithelium. CK 20 is commonly found in colorectal and gastric cancer, transitional cell carcinomas and in Merkel cell carcinomas. Non-mucinous ovarian cancer does not express CK 20. It is often used in combination with CK 7 to distinguish different types of glandular tumors.

Studies conducted in recent years have focused on a search for an immunohistochemical marker to aid the differential diagnoses of primary and metastatic ovarian carcinoma. Our study objective was to study the usefulness of Wilms tumor 1 (WT 1) antigen in this context. WT 1 is a tumor supressor gene (TSG) located on chromosome 11. It has a profound role in genitourinary system development. Unlike other TSGs, WT 1 expression is also found in normal human cells such as mesothelium and fallopian tube epithelium. WT 1 protein can be demonstrated in most ovarian serous carcinomas as well allowing these tumors to be distinguished from other adenocarcinomas. WT 1 also may have a cross reaction with cytoplasmic proteins, so only nuclear staining is considered diagnostic.

## MATERIALS AND METHODS

### a. Subject specimens

The study was conducted at the pathology clinic of Lütfi Kırdar Training and Research Hospital. Approval was obtained from the hospital’s review board. Deparaffinated blocks of 40 epithelial ovarian tumors and 40 colon adenocarcinomas were studied. In addition, 35 blocks of omental metasteses were included with the aim of determining the primary origin. CK 7, CK 20, and WT 1 were applied to all specimens. Afterwards, archive records of cases with omental metastases were sought and primary disease locations were revealed.

### b. Immunohistochemistry

Three-micron-thick sections were cut from the paraffin blocks and placed on poly L-lysine- coated slides and stored in an oven at 37 ºC overnight. The slides were passed through series of alcohol dilutions for 15 minutes each followed by distilled water for rinsing. For antigen retrieval, slides were placed in a plastic coplin jar filled with citrate buffer (pH 6-0) and covered with perforated cling film to minimize evaporation, and then placed in a microwave oven and irradiated at 800 W, 600 W and 360 W, respectively, for five minutes each. Slides were allowed to cool at room temparature for 20 minutes and rinsed. Endogenous peroxidase was blocked (Novacastra protein block RE 7102, Lot 710257). The slides were then rinsed with phospate-buffered saline. The treated slides were immunostained with Wilms Tumor Monoclonal Mouse anti-Human (Leica Band Wilms’ Tumor WT 49, 7 mL), CK 7 Monoclonal Mouse anti-Human (NCL-L-CK 7-560 Novacastra 1: 100; Lot: L156019), and CK 20 Monoclonal Mouse anti-Human (NCL-L-CK 20 Novacastra 1: 50; Lot: 6000573). Diaminobenzidine chromogen system was applied on slides for five minutes in order to observe the immune reaction. All slides were rinsed and contrast stained with Meyer’s hematoxylin and cleared with xylene. CK 7 and CK 20 expression was cytoplasmic and WT 1 expression was nuclear.

## RESULTS

Forty clinically diagnosed cases of ovarian carcinoma, 40 colonic adenoarcinomas, and 35 omental metasteses were examined. The median age of patients with omental metastases was 63.26±11.2 years, with ovarian carcinoma was 53.68±11.68 years, and colonic adenocarcinoma was 59.13±14.30 years.

**Figure 1:**
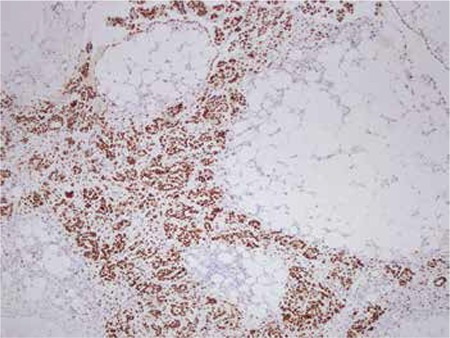
Omental metastasis of primary ovarian carcinoma stained with WT 1

Primary ovarian adenocarcinomas stained positive with WT 1 in 82.5% of the cases and all colorectal adenocarcinomas were negative with WT 1 (100%) (Table 1). Of the 35 cases of omentum metastases, 54.3% stained positively with WT 1 (Figure 1).

**Figure 2:**
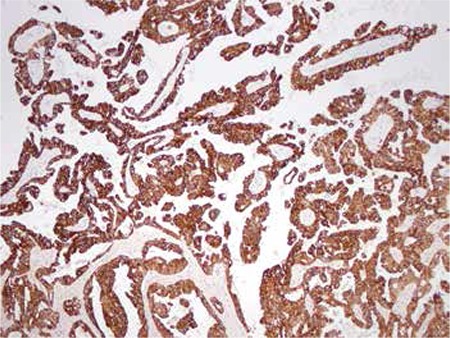
Serous ovarian carcinoma stained with cytokeratin CK 7

All ovarian adenocarcinomas stained positively with CK 7 (100%) (Figure 2), whereas 92.5% of colorectal adenocarcinomas were negative with CK 7. Of the 35 cases of omentum metastases, 60% stained positively with CK 7.

**Figure 3:**
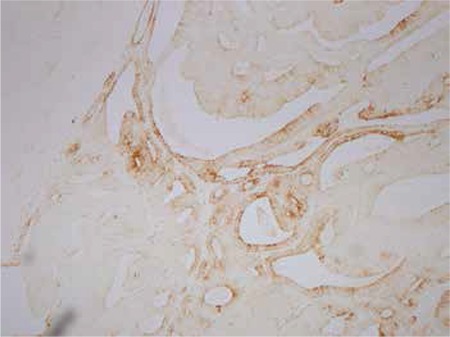
Colon adenocarcinoma stained with cytokeratin 20

Primary ovarian adenocarcinomas stained positively with CK 20 in 7.5% (n=3) of cases. These 3 cases were revealed to be mucinous in origin. Colorectal adenocarcinomas stained positively with CK 20 in 87.5% of cases (Figure 3). Out of the 5 specimens of colorectal adenocarcinomas that did not stain, one was of mucinous origin. Of the 35 cases of omentum metastases, 40% stained positively with CK 20.

Archive records revealed that out of the 35 cases of omentum metastases, 22 were ovarian and 13 were of colorectal origin. Nineteen (86.4%) of the 22 ovarian metastases stained positively with WT 1. None of the metastases of colorectal origin stained with WT 1. Twenty-one (95.5%) of the 22 ovarian metastases stained positively with CK 7. None of the metastases of colorectal origin stained with CK 7. Eleven (50%) of the 22 ovarian metastases stained positively with CK 20, along with 10 (76.9%) cases of metasteses of colorectal origin (Table 2).

Five out of the 40 primary ovarian carcinomas were of mucinous origin. These carcinomas showed differences in staining. In three cases, a CK 7+, CK 20+, and WT 1- staining pattern was seen, one was CK 7+, CK 20+, and WT 1+, and the remainder did not stain with any of the markers.

Fifteen out of the 40 primary colorectal carcinomas were of mucinous origin and none of these expressed WT 1 or CK 7 (Table 3). All but one (93.3%) stained positively with CK 20.

## DISCUSSION

Various studies have shown that metastatic ovarian carcinomas generally stain positively with CK 7 and negatively with CK 20, whereas the opposite is true for colorectal carcinomas. However, mucinous carcinomas frequently express both antigens and can present a diagnostic challenge^(10,11)^. Groisman et al.^(12)^ researched CdX2 in order to aid the differential diagnosis of primary and secondary colorectal adenocarcinomas. The authors suggested that CdX2 was more specific than CK 20 for colorectal adenocarcinoma and that inclusion of CdX2 in antibody panels to distinguish between primary and secondary epithelial colorectal malignancies may be helpful.

In our study, 87.5% of primary colorectal adenocarcinomas and 76.9% of colorectal omental metastases stained positively with CK 20. These percentages are lower than Groisman’s results but we believe that mucinous carcinomas accounted for the discrepancy^(12)^. Ovarian carcinomas did not stain with CK 20 in 92.5% of the cases. Whilst ovarian nonmucinous adenocarcinomas do not express CK 20, CK 20 expression of mucinous ovarian carcinomas has been studied. Loy et al.^(13)^ reported 60% positive staining results with CK 20 in serous ovarian carcinomas. In contrast, Berezowski et al.^(10)^ showed that differentiated colorectal adenocarcinomas did not show a positive CK 20 staining pattern. In the above mentioned study, Groisman was searching for a more specific marker than CK 20 for colorectal carcinoma. In our study, we had a similar aim to introduce WT 1 in an antibody panel for ovarian carcinoma. All of the ovarian adenocarcinoma slides stained positively with CK 7 and 82.5% stained with WT 1. However, CK 7 expresion can also be seen with gastrointestinal, lung, and breast adenocarcinomas. These results have led us to the possibility of using these markers in adjunction.

Ordenez^(14)^ applied WT 1 staining to 135 adenocarcinomas including ovarian, colorectal, renal, thyroid, and prostate origin. Among the adenocarcinomas, only ovarian adenocarcinomas expressed WT 1. Loeb et al.^(15)^ used western blotting as well as immunohistochemistry and showed WT 1 positivity in 27 out of 31 breast adenocarcinomas. As a result of their study, WT 1 was questioned as an oncogene for breast cancer rather than a tumor supressor gene. Miyoshi et al.^(16)^ proposed that high expression of WT 1 was a poor prognostic factor for breast adenocarcinoma. Other researchers have suggested that altered expression of WT 1 has a role in breast cancer development^(17)^. Yet, other researches concluded with contradictory results. Harry Hwang et al.^(18)^ applied WT 1 to 118 breast cancer tissue specimens and yielded postive results in only 8 cases. Inoue et al.^(19)^ showed that WT 1 was a prognostic marker for leukemia. Hylander et al.^(20)^ demonstrated that WT 1 played a prognostic role in ovarian adenocarcinoma and that its expression was correlated with tumor grade and stage but not with survival. Another study of WT 1 on ovarian tissue by Shimizu et al.^(21)^ found positive WT 1 staining with ovarian surface epithelium, inclusion cysts, and fallopian tubes, but not with cervical or endometrial epithelial tissue.

In our study we have shown that CK 7 was a more sensitive marker than WT 1 for ovarian carcinoma; however, CK 7 expression is frequently seen in the gastrointestinal system, lung, and breast tumors. We can hypothesise that WT 1 can be used beacuse our study showed that 82.5% ovarian tumors and none of the colerectal carcinomas expressed WT 1.

To summarize, CK 20 does not differentiate mucinous or nonmucinous colorectal carcinoma. Regarding mucinous tumors, our markers CK 7, CK 20, and WT 1 were not useful. WT 1 can be used in conjuction with CK 7 in the differential dianosis of ovarian carcinomas.

## Figures and Tables

**Table 1:**
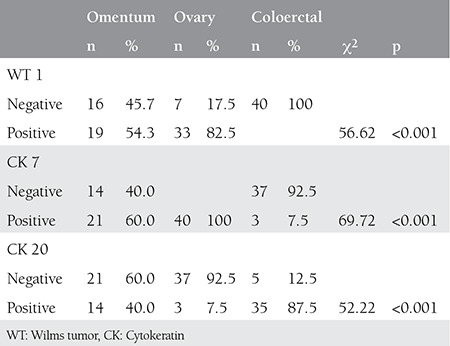
Immunohistochemistry staining of the specimens

**Table 2:**
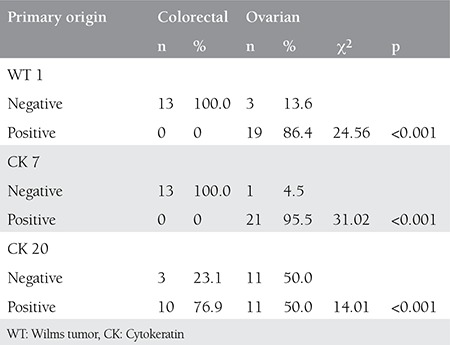
Immunohistochemistry staining of omental metastases and their primary origins

**Table 3:**
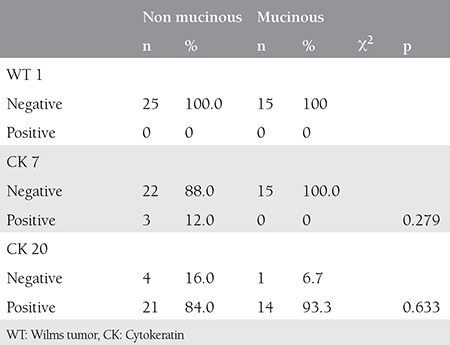
Staining patterns of colorectal adenocarcinomas
